# Intramuscular hemangioma presenting as ankle equinus deformity in a pediatric patient: a case report and literature review

**DOI:** 10.1093/jscr/rjaf847

**Published:** 2025-10-23

**Authors:** Khalid Bakarman, Mishari Alanezi, Nouf Alabdulkarim, Abdullah S Alzaid, Aref A Altawair

**Affiliations:** Department of Orthopedic Surgery, College of Medicine, King Saud University, PO Box 2925, Riyadh 11461, Saudi Arabia; College of Medicine, King Saud University, PO Box 2925, Riyadh 11461, Saudi Arabia; Department of Orthopedic Surgery, College of Medicine, King Saud University, PO Box 2925, Riyadh 11461, Saudi Arabia; Department of Orthopedic Surgery, College of Medicine, King Saud University, PO Box 2925, Riyadh 11461, Saudi Arabia; Al Yamamah Hospital for Children and Maternity, PO Box 2925, Riyadh 14222, Saudi Arabia

**Keywords:** Z-lengthening, hemangioma, sclerotherapy, pediatric

## Abstract

Ankle equinus deformity is characterized by limited ankle dorsiflexion, causing gait disturbance, pain, and functional limitations. Common causes include congenital anomalies, cerebral palsy, and muscular dystrophy, while intramuscular hemangiomas (IMHs) are extremely rare. The patient, a 7-year-old girl, presented with right ankle equinus and toe-walking, first noted at age 5, as a persistent, non-tender swelling in the posterior leg. Examination showed fixed ankle equinus, asymmetric calf circumference, and a 1 cm leg length discrepancy. Magnetic resonance imaging (MRI) revealed a large IMH involving the gastrocnemius muscle. She underwent five sessions of sclerotherapy over 18 months, followed by open Achilles tendon Z-lengthening. At 2-year follow-up, she had a painless plantigrade foot with 20° dorsiflexion and was independently ambulating, indicating successful functional recovery. This case highlights the rare presentation of ankle equinus due to IMH. MRI is essential for diagnosis, and combined sclerotherapy with tendon lengthening can achieve excellent outcomes.

## Introduction

Ankle equinus deformity is characterized by limited dorsiflexion, leading to gait disturbance, pain, and functional limitation [1]. It can have various etiologies, including congenital anomalies and acquired conditions. Acquired etiologies include cerebral palsy, trauma, muscular dystrophy, poliomyelitis, and rarely intramuscular hemangiomas (IMHs) [2].

Hemangiomas are benign proliferative vascular tumors that account for 7% of all soft tissue tumors [3]. In particular, IMHs are extremely rare, as they account for 0.8% of all hemangiomas, where muscles are invaded, causing contracture and shortening [4]. IMHs commonly arise in the lower extremities, particularly the thigh and calf muscles. When located in the calf, they involve the gastrocnemius and soleus muscles, leading to progressive contracture and soft tissue fibrosis, resulting in limited ankle dorsiflexion and contributing to equinus deformity and gait disturbance [5]. IMHs are often asymptomatic and discovered incidentally, although they can cause pain and swelling, especially after exercise, due to vascular dilation and increased blood flow [6]. Early diagnosis and appropriate management are crucial to prevent functional impairment. In this case report, we describe a rare presentation of ankle equinus deformity secondary to IMH in a 7-year-old girl. The patient's guardian voluntarily agreed to publish this report for educational purposes, and informed consent was obtained.

## Case report

A 7-year-old girl presented to our facility with a history of right ankle equinus deformity and toe-walking, which were first noted at the age of 5 years, accompanied by persistent swelling in the posterior aspect of the ipsilateral leg. The swelling remained stable in size and was initially evaluated at a secondary hospital, where magnetic resonance imaging (MRI) suggested a benign soft-tissue lesion.

**Figure 1:**
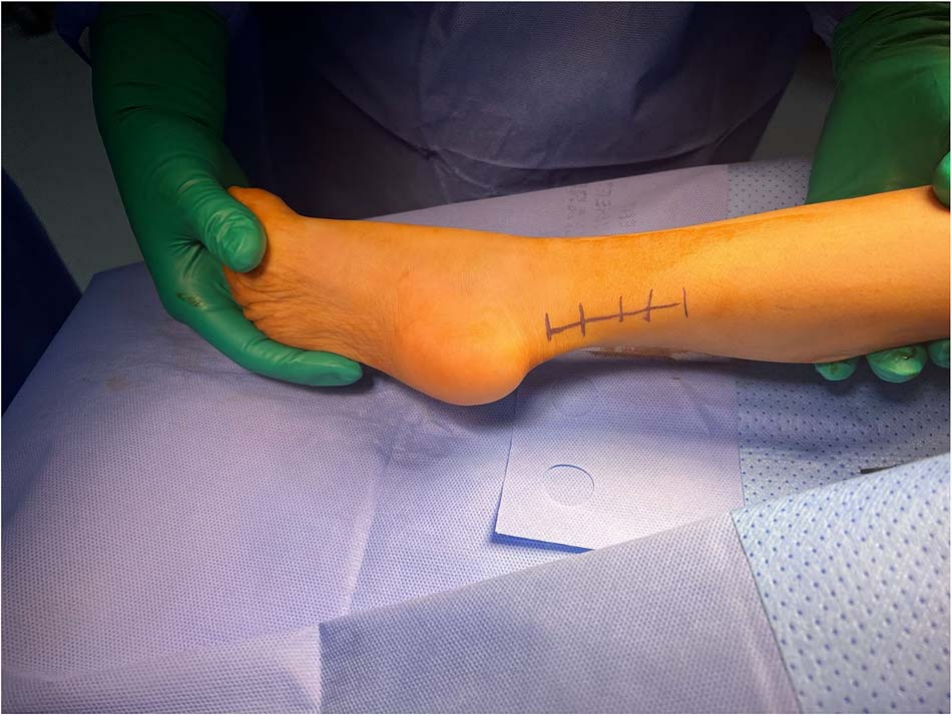
Maximum dorsiflexion before Achilles tendon Z-lengthening.

**Figure 2:**
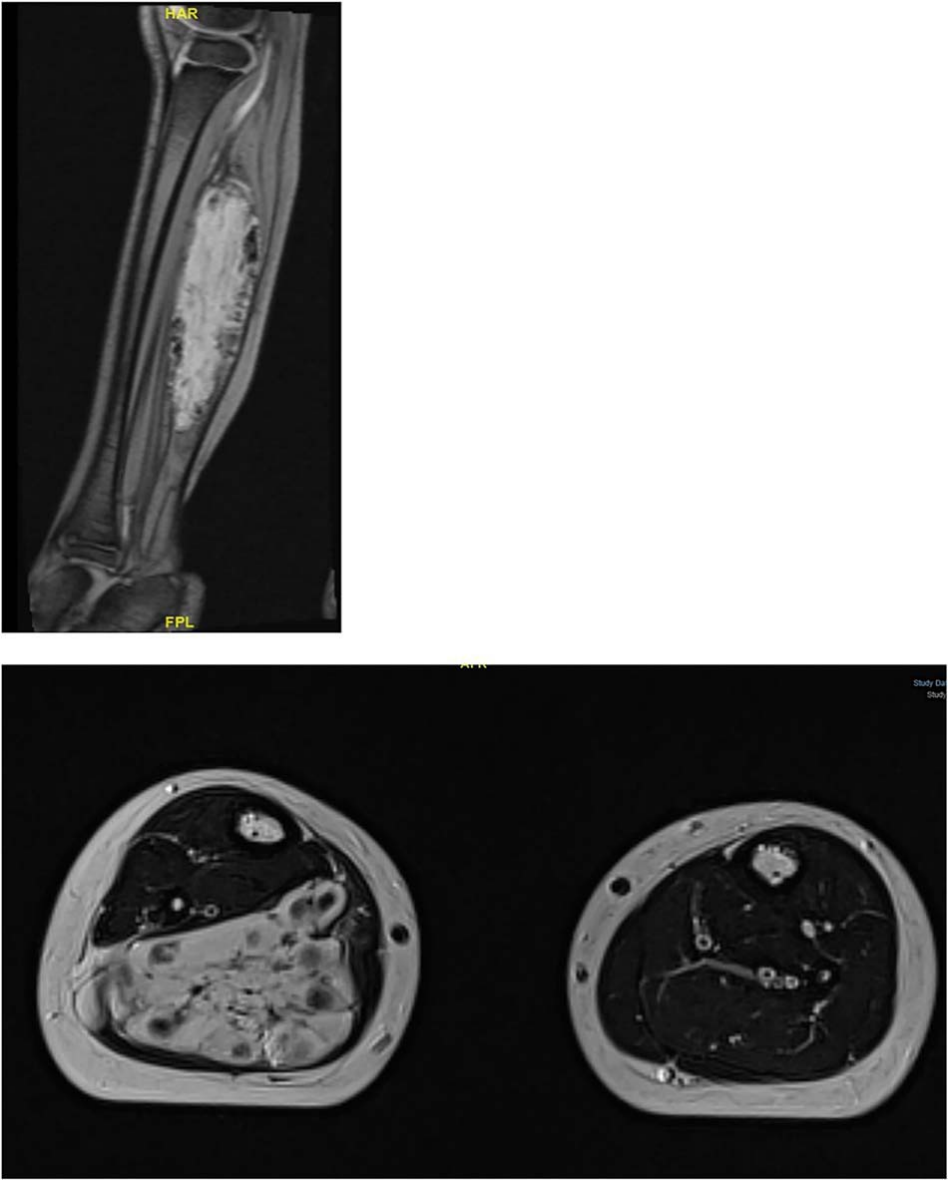
Large, lobulated intramuscular soft tissue vascular lesion with multiple calcified phleboliths, suggesting intramuscular hemangioma.

Physical examination revealed a tip-toe gait with a fixed 35° right ankle equinus deformity (Fig. 1). A firm, non-tender swelling measuring 7 × 3 cm was observed in the posterior mid-leg. Asymmetric leg circumferences were noted (right: 22 cm, left: 20 cm), along with a 1 cm leg length discrepancy, which was confirmed radiologically. Radiographic imaging of the right leg showed a posterior soft tissue shadow with areas of ectopic calcification. Contrast-enhanced MRI revealed a large, lobulated soft tissue lesion measuring 5 × 2.8 × 10 cm, located deep within the calf muscles (Fig. 2). The lesion contained multiple calcified phleboliths and exhibited an isointense signal on T1-weighted images with high signal intensity on T2-weighted images, consistent with an IMH.

**Figure 3:**
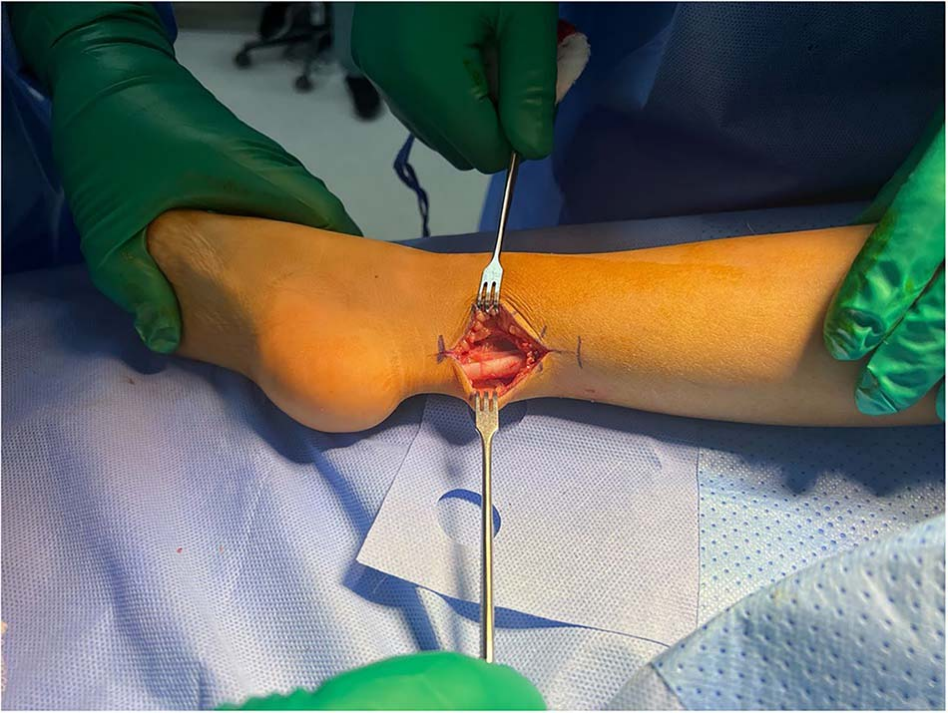
Achilles tendon exposed for Z-lengthening.

**Figure 4:**
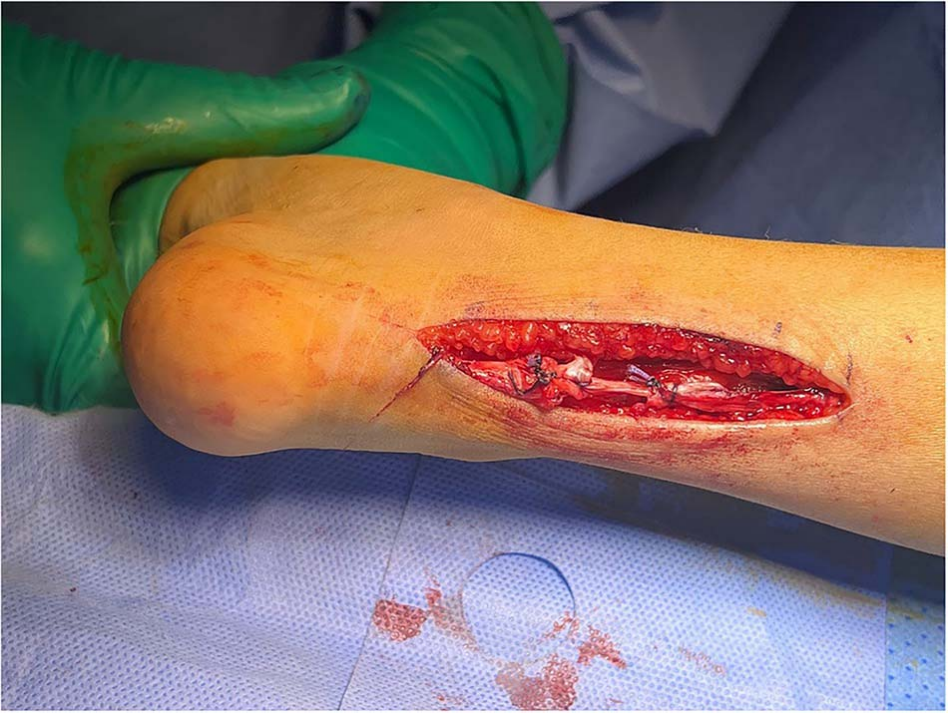
Tendoachillis after lengthening, showing improved dorsiflexion.

**Figure 5:**
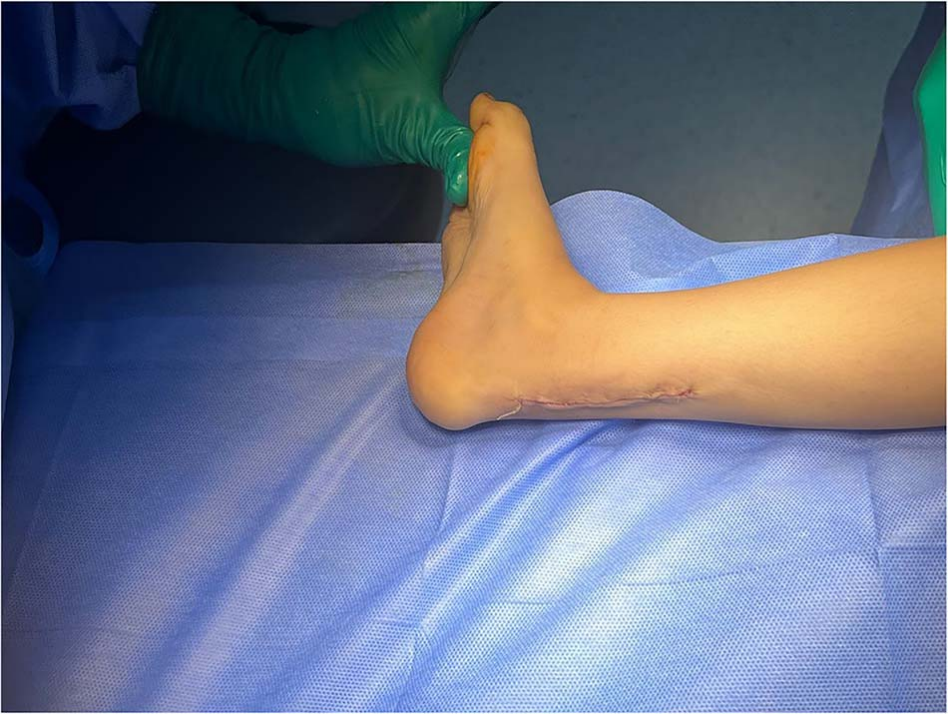
After lengthening passive dorsiflexion reaching 20°.

The patient underwent five sessions of sclerotherapy over 18 months, followed by open Achilles tendon Z-lengthening (Figs 3 and 4). During the follow-up period, her dorsiflexion angle gradually improved with physical therapy (Fig. 5). At the 2-year follow-up, she demonstrated a painless plantigrade ankle with 20° dorsiflexion, indicating a successful outcome.

## Discussion

Hemangiomas are benign vascular tumors characterized by abnormal proliferation of endothelial cells. While hemangiomas commonly affect the skin and subcutaneous tissue, IMHs are an extremely rare subset, accounting for less than 1% of all hemangiomas [4, 7].

IMHs predominantly affect adolescents and young adults, with more than 80% presenting before the age of 30 years and a male-to-female ratio of 1.4:1 [8]. Patients with IMHs are often asymptomatic; however, pain, swelling, and discomfort are common after physical exercise [6].

IMHs predominantly involve the lower extremities, most commonly the thigh (36%), followed by the calf muscles (17%) [9]. Unlike cutaneous hemangiomas, they do not regress spontaneously and may cause progressive pain, palpable masses, and muscle contractions, leading to deformities [10]. In the lower extremities, particularly the calf muscles, progressive fibrosis and contraction can restrict ankle dorsiflexion, resulting in ankle equinus deformity [5].

Diagnosis of IMHs can be challenging because of their deep location and nonspecific symptoms. A thorough clinical evaluation, including detailed history, physical examination, and radiological imaging, is crucial for an accurate diagnosis [7]. Plain radiographs are often inconclusive, although faint calcifications are seen in 15% of patients [11]. Contrast-enhanced computed tomography (CT) is useful for assessing the vascularity of lesions. While radiographs and CT are useful initially, MRI is the preferred modality, as it provides detailed information on the lesion’s size, extent, relationship with surrounding structures, and signal intensity [12].

The management of IMHs depends on several factors, including the lesion’s size, location, symptom severity, and functional impairment. Asymptomatic or minimally symptomatic lesions may be managed conservatively with regular follow-up and nonsteroidal anti-inflammatory drugs for pain relief. However, for severely symptomatic lesions or lesions causing substantial functional limitations, more definite interventions are required [13]. Minimally invasive interventions, including sclerotherapy and embolization, are preferred for lesions that are not surgically accessible.

Sclerotherapy involves injecting a sclerosing agent directly into the hemangioma to induce fibrosis, leading to lesion shrinkage. It is less invasive than surgical excision, with a reported cure and marked improvement rate of ~70% [14]. Embolization is another minimally invasive intervention that occludes the primary blood supply to the lesions, resulting in regression [15]. Despite the usefulness of minimally invasive techniques, surgical excision remains the mainstay of treatment. Complete surgical excision of the hemangioma with the surrounding margin of normal muscles is considered the optimal option with the lowest risk of recurrence, although a large lesion size and incomplete marginal resection are associated with a recurrence rate.

Ankle equinus deformity secondary to a vascular anomaly in the calf muscles is exceptionally rare. In this case report, we present the rare case of a 7-year-old girl with ankle equinus deformity secondary to a gastrocnemius hemangioma who was treated with sclerotherapy and open Achilles tendon lengthening. Postoperatively, the patient had an excellent functional outcome and was ambulating independently during routine surveillance follow-up visits at 3, 6, and 9 months as well as at 2 years.

## Data Availability

All data are available upon request for the corresponding Author.
